# Supplementary material for: Cu-Doped MnO2 Catalysts for Effective Fruit Preservation via Ozone Synergistic Catalytic Oxidation
Source: Foods. 2024 Dec 20;13(24):4127. doi: 10.3390/foods13244127 (PMC11675573; doi:10.3390/foods13244127)
Supplement: Supplementary file 1 [file foods-13-04127-s001.zip › foods-3310375-supplementary.pdf]

Cu-doped MnO_2 catalysts for effective fruit preservation via ozone synergistic catalytic oxidation

JianGuo Huang[1], Rashid Khan[2], Chunhui Zhai[3,4], Xianting Ding[3,4], Li-Sha Zhang[1], Jin-Ming Wu*[1], Zhizhen Ye[1]

[1] State Key Laboratory of Silicon and Advanced Semiconductor Materials, School of Materials Science and Engineering, Zhejiang University, Hangzhou, 310027, P. R. China.

[2] Zhejiang Provincial Key Laboratory of Advanced Chemical Engineering Manufacture Technology, College of Chemical and Biological Engineering, Zhejiang University, Hangzhou 310027, PR China.

[3] Department of Anesthesiology and Surgical Intensive Care Unit, Xinhua Hospital, School of Medicine and School of Biomedical Engineering, Shanghai Jiao Tong University, Shanghai, China

[4] State Key Laboratory of Oncogenes and Related Genes, Institute for Personalized Medicine, Shanghai Jiao Tong University, Shanghai, China

*Corresponding author: msewjm@zju.edu.cn (Jin-Ming Wu)

Antibacterial test

The step-wise procedure for the antibacterial test is outlined as follows:

(1) Bacterial preparation: The 3rd to 7th generation of test bacteria (E. coli) were freshly cultured for 24 h at 36 ± 1°C on agar slants. The bacterial lawn was washed off using saline solution containing peptone and then filtered through sterile cotton wool. Subsequently, the sample was diluted to the required concentration using a nutrient broth medium.

(2) Chamber conditioning: Simultaneously adjust the temperatures of the two test chambers to 25°C with a relative humidity of 70%.

(3) Chamber initialization: Activate the chamber controller, stirring device, and lighting. Henceforth, all operations and equipment manipulations will be conducted externally via remote control. The chamber door was opened only upon the completion of the test.

(4) Sample application: Apply 1 mL of the bacterial solution evenly across six nutrient agar plates. These inoculated plates were divided into two sets and placed into the control and test chambers. In the test chamber, activate the internal fan and ozone generator for disinfection.

(5) Post-disinfect evaluation: After 12 h of disinfection, the nutrient agar plates were removed from both the control and test chambers. Seal the plates and incubate them upside down in an oven set at 36°C for 24-48 h. Subsequently, the bacterial colonies were counted to evaluate the disinfection efficacy.

XPS results of Cu-doped δ-MnO_2

The chemical states of the Cu-doped δ-MnO_2 catalyst was also investigated by XPS. The survey spectrum contained Cu, Mn, and O elements, showing that Cu atoms were successfully incorporated in the δ-MnO_2 catalyst as well, as can be seen in figure S1(a). The high-resolution spectrum of Cu 2p (Figure S1(b)), where the two peaks at binding energies of 933.9 and 952.6 eV corresponded to the Cu $2p_{3/2}$ and Cu $2p_{1/2}$ electronic states, respectively, suggesting that copper is primarily in the divalent valence state. The high-resolution Cu 2p spectrum also contained two satellite peaks located at 944.9 and 960.3 eV [1]. The Mn 2p high-resolution XPS spectrum exhibited two major peaks at 642.1 and 653.9 eV associated with the valence states of Mn $2p_{3/2}$ and Mn $2p_{1/2}$ (Figure S1(c)), respectively. The valance state of Mn $2p_{3/2}$ can be deconvoluted into two peaks located at 642.1 and 643.4 eV, attributed to Mn^{3+} and Mn^{4+}, respectively [2]. The content of Mn^{3+}, Mn^{4+}, and Mn^{3+}/Mn^{4+} is listed in Table S2. The addition of copper in the catalyst could replace Mn^{4+} or Mn^{3+} ions and increase the oxygen vacancies and causing imbalance in the system. The spin energy difference between the two major peaks of Mn 2p was about 11.8 eV. The O 1s high-resolution spectrum (Figure S1(d)) exhibited three peaks at 529.4, 531.1 and 532.2 eV corresponded to lattice oxygen (O_{latt}), oxygen vacancy defect (O_v), and surface adsorbed water (O-H), respectively.

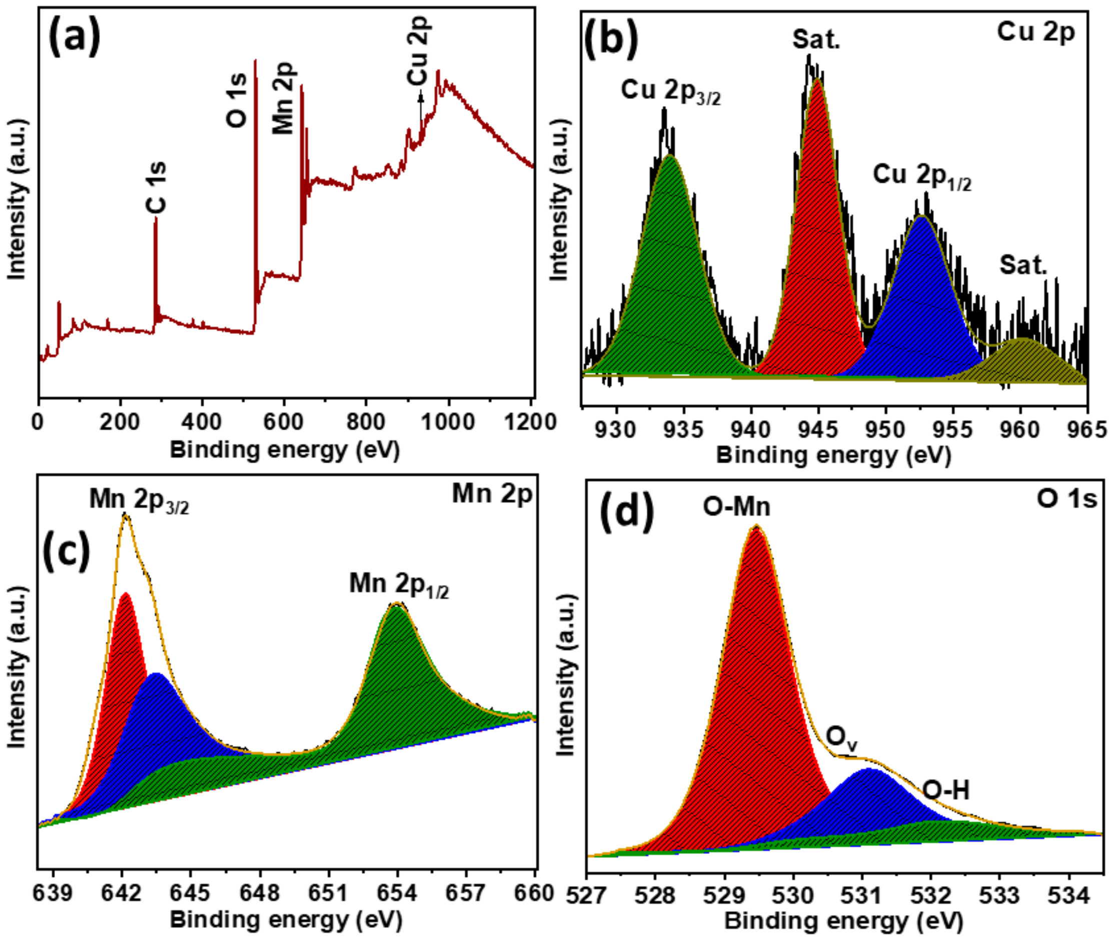

Figure S1. XPS analysis of Cu-doped δ-MnO_2 catalyst: (a) survey, (b) high-resolution spectra of Cu 2p, (c) Mn 2p, and (d) O 1s.

Table S1. Mn^{3+} and Mn^{4+} peaks percentage calculated by fitting of Mn $2p_{3/2}$ peak and oxygen vacancy percentage calculated by fitting of O 1s spectra.

Samples	Mn^{3+}	Mn^{4+}	Mn^{3+}/ Mn^{4+}	Oxygen Vacancy (O_v)
Cu-doped α-MnO_2	65.13%	34.87%	1.86	31.62%
Cu-doped δ-MnO_2	52.89%	47.11%	1.12	21.45%

Ethylene degradation experiment

The ethylene degradation experiment was conducted using two identical test chambers with a size of 1 m^3, serving as the experimental and control chambers, at a temperature of 25 ± 1 °C, humidity of 70 ± 5%, and ozone concentration of 40 ppb. Each chamber contained ten bananas and five apples and the concentration of ethylene inside the box was measured using an ethylene detector (NEO, MP-180). Cu-doped MnO_2 catalysts with different crystal phases were utilized for the degradation of ethylene, and the OSCO technology chamber contained 275 g of catalyst-soaked honeycomb with a total volume of 327 cm^3 (Figure S2(a)). The airflow rate was set at 35 m^3/h. In contrast, the control setup did not include any catalyst-soaked honeycomb but maintained ozone concentrations below 40 ppb (Figure S2(c)). Every 24 h, the ethylene concentration inside the testing chamber was read remotely for up to ten days using an ethylene detector.

As illustrated in Figure S2(b), bananas treated with OSCO technology were fresher than those untreated with OSCO (Figure S2(d)). The ripening phase is characterized by the appearance of dark and tan markings that result from enzymatic browning. The phenomenon occurs when phenolic substances exit the cell and undergo oxidation by the enzyme polyphenol oxidase (PPO) [3]. The pigment responsible for the dark and brown coloration was formed when the quinone compound was polymerized. The Cu-doped α-MnO_2 catalyst acted as an ethylene scavenger and effectively slowed the ripening of bananas and apples. The main objective of this study was to support preservation efforts at the retail level, where bananas and apples undergo artificial ripening. At this point, in the rapid maturation process, postponing sales by just one day could potentially yield financial benefits for the vendor. Therefore, OSCO technology has proven to be more effective in preserving fruits than ozone alone.

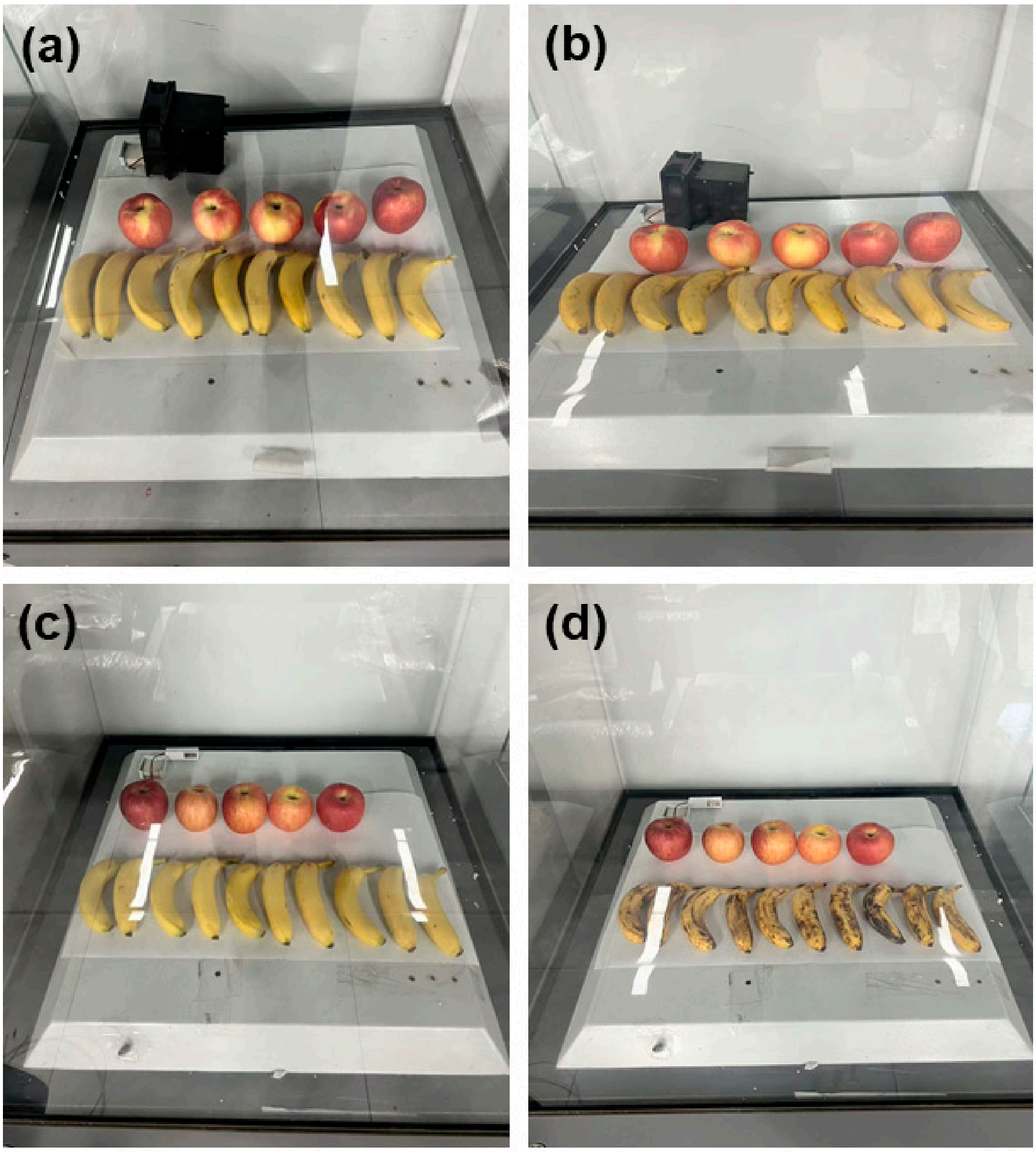

Figure S2. Degradation of ethylene using OSCO technology (a) Ethylene degradation using OSCO technology, (b) Fruits picture after ten days, (c) Ethylene degradation without OSCO technology and (d) Fruits picture after ten days.

References

[1] Wang, G., Wang, Y., Guan, B., Liu, J., Zhang, Y., Shi, X., Tang, C., Li, G., Li, Y., Wang, X. and Li, L., 2021. Hierarchical K-birnessite-MnO_2 carbon framework for high-energy-density and durable aqueous zinc-ion battery. *Small*, *17*(45), p.2104557.

[2] Yang, W., Su, Z.A., Xu, Z., Yang, W., Peng, Y. and Li, J., 2020. Comparative study of α-, β-, γ-and δ-MnO_2 on toluene oxidation: Oxygen vacancies and reaction intermediates. *Applied Catalysis B: Environmental*, *260*, p.118150.

[3] Nugroho, F.G., Agson-Gani, P.A., Anindita, P.A., Steky, F.V., Benu, D.P., Yuliarto, B., Dwivany, F.M. and Suendo, V., 2024. Prolonging banana shelf life through visible light-induced ethylene scavenging using manganese-decorated TiO_2 via $KMnO_4$ reduction. *Colloids and Surfaces A: Physicochemical and Engineering Aspects*, *691*, p.133817.
